# Primary Percutaneous Coronary Intervention for Acute Myocardial Infarction in a Young Female with Idiopathic Thrombocytopenic Purpura: A Case Report and Review 

**DOI:** 10.1155/2010/854682

**Published:** 2010-03-24

**Authors:** Ahmet Yildiz, Ugur Coskun, Ozlem Esen Batukan, Kudret Keskin

**Affiliations:** Institute of Cardiology, Istanbul University, Adıvar cad. 34096 Haseki, Istanbul, Turkey

## Abstract

A 23-year-old female with the diagnosis of idiopathic thrombocytopenic purpura (ITP) was admitted to our hospital with severe chest pain. The electrocardiogram (ECG) revealed acute anterior myocardial infarction. She underwent an immediate cardiac catheterization. An occluded left anterior descending (LAD) was detected by coronary angiography. Reperfusion was performed successfully by angioplasty and stenting with optimal distal flow without any complications. In this report we discussed the management strategies of acute myocardial infarction (AMI) in a patient with ITP.

## 1. Introduction

Idiopathic thrombocytopenic purpura (ITP) is an autoimmune disorder characterized by a low platelet count and mucocutaneous bleeding [1]. Since the prevalence of ITP is 9.5 per 100 000 persons, acute myocardial infarction (AMI) patients with ITP are seen very rare [2–4]. Thrombolytic therapy is contraindicated in ITP patient with AMI. However, a few cases of primary percutaneous coronary intervention (PCI) for AMIs have been reported in patients with ITP [3–7].

## 2. Case Report

A 23-year-old female was admitted to our hospital with severe chest pain. The patient had been diagnosed with chronic ITP 4 years ago. She was treated with steroids which had been stopped one year previously. Two weeks prior to admission, she was started on a course of steroids due to a low platelet count. 

**Figure 1:**
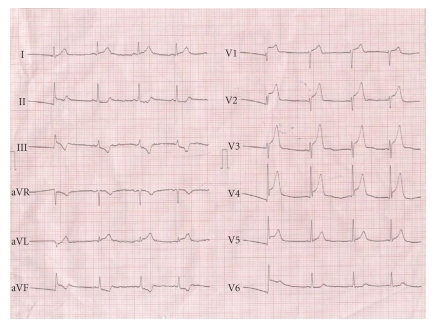
Electrocardiogram presents ST segment elevation in leads of V2–V5 and aVL.

**Figure 2:**
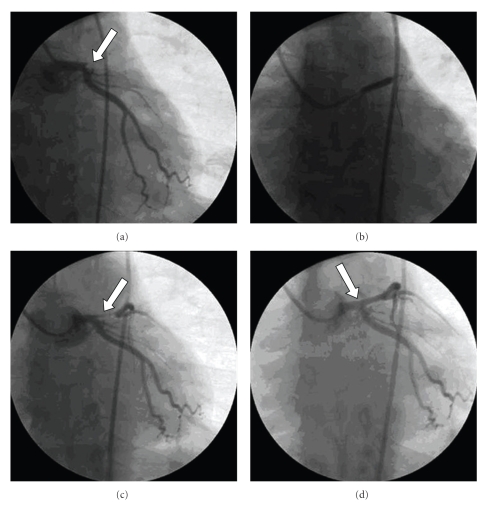
Coronary angiography of the left coronary artery. (a) Total obstruction in the proximal left anterior descending artery (LAD). (b) Dilation. (c) After balloon angioplasty. (d) After stent implantation, PCI was successful.

On admission, the electrocardiogram (ECG) revealed a regular sinus rhythm with a rate of 80/min and ST segment elevations in V2–V5 and aVL (Figure 1). The platelet count was 35 000/*μ*L. The patient was immediately transferred to the catheterization laboratory. Angiography revealed total occlusion of the left anterior descending artery (LAD; Figure 2). The circumflex and right coronary arteries were normal. Then, 600 mg of clopidogrel was loaded and weight-adjusted UFH (60 U/kg) was administered intravenously just before the PCI. After crossing the total occlusion with a 0.014 inch high-torque floppy wire the lesion was dilated with a 2.0 × 20 mm balloon (Ma'slaJut, Asahi Intecc, Japan) with an inflation pressure of 12 atm. After pre-dilation, a 3.0 × 20 mm Mi-JAZZ stent was implanted at 12 atm pressure (bare metal; Millimed, Raskijde, Denmark) into the LAD.

Subsequent angiography demonstrated thrombolysis in myocardial infarction (TIMI) grade III flow and optimal myocardial contrast blush (Figure 2). After restoration of blood flow, the chest pain resolved and the ST segment elevations in the ECG regressed within 1 hour. The activated partial thromboplastin time was 155 seconds. Following the procedure, the patient was transferred to the intensive care unit (ICU) and treated with aspirin (300 mg/day), clopidogrel (75 mg/day), metoprolol (50 mg/day), ramipril (2.5 mg/day), and atorvastatin (40 mg/day); heparin was not administered. The sheath introducer was removed from the right femoral artery after the PCI, and hand compression was applied to the puncture site for more than 30 minutes to achieve complete hemostasis. We noted ecchymoses around the puncture site of the right femoral artery. No other bleeding complications occurred. The patient was subsequently monitored in the ICU for 48 hours and discharged after 5 days without any complications related to the myocardial infarction and the PCI.

## 3. Discussion

ITP is characterized by the presence of antiplatelet antibodies, immune platelet destruction, and a low platelet count [8]. An AMI is rarely experienced in patients with ITP [2]. Only five cases of primary PCI for AMI in patients with ITP have been reported in the literature ([3–7]; Table 1). The mean age of these 5 patients was 65 years; therefore, our patient is the youngest patient to be reported in the literature. ITP occurs with a higher incidence (3  :  1) among female adult patients [1].

Fuchi et al. [3] reported a 72-year-old female diagnosed with ITP in 1999. She had never smoked, and there was no history of ischemic heart disease in her family. She had a history of hyperlipidemia. Coronary angiography revealed total occlusion of the LAD. Although PCI was successfully performed for stenosis in the proximal LAD, the condition of the patient was complicated by a reinfarction. A large hematoma was observed around the femoral artery puncture site. For this reason, immunoglobulin and methylprednisolone were administered intravenously.

Kikuchi et al. reported a case of acute anterior myocardial infarction in a 68-year-old woman with hypertension, hyperlipidemia, and diabetes mellitus and a platelet count of 22,000/*μ*L [4]. PCI was performed and there was no bleeding tendency with heparin infusion and ticlopidine hydrochloride.

Another case of coronary involvement presenting with an inferior myocardial infarctioninvolved a 47-year-old woman with chronic ITP. Heparin was administered during PCI only, and combined antiplatelet therapy (aspirin 200 mg and clopidogrel 75 mg) was performed. No major bleeding complications occurred [5]. 

Fong et al. [6] reported a 71-year-old ITP patient who presented with acute coronary syndrome. Cardiac catheterization was performed through the radial artery with the administration of immunoglobulin premedication. This was followed by clopidogrel treatment for 7 weeks without major bleeding complications. Gracia et al. [7] recently reported a young man who had total LAD occlusion and was treated successfully by primary PCI under cover of unfractionated heparin prophylaxis and anti-aggregation with clopidogrel and ASA. 

If patient with ITP has coronary artery disease, concomitant influence of traditional coronary risk factors should be considered. In our case, the patient did not have any known coronary artery risk factors, such as a family history of coronary artery disease, hypercholesterolemia, hypertension, diabetes, or smoking. She also had not had arteritis, trauma, embolization (factor V, factor VIII, and total homocysteine level), dissection, spasm, or congenital anomalies. We thought that drug use (steroids) was the most probable etiology in our case. Steroids are known to induce metabolic changes, as well as a hypercoagulable state [9]. 

Another hypothesis is that the pathogenesis of myocardial infarction in thrombocytopenic patients with ITP may result from endothelial damage induced by autoantibodies directed against antigens, which are present in both platelets and coronary endothelial cells [10].

Transradial approach of coronary angiography and angioplasty has become a good alternative to the femoral approach in all patients due to the low rate of access site complications, improved patient comfort, and early ambulation. This approach is particularly applicable to patients receiving anticoagulation treatment or thrombolytics due to the ease of compressing the puncture site. 

The other important issue for ITP patients is control of hemostasis. Fuchi et al. [3] reported that large hematomas were observed around the puncture sites. Sheath introducers from the radial artery can be removed after the procedure, followed by compression to achieve complete hemostasis of the puncture sites. After the procedure, intravenous immunoglobulin can be used to prevent bleeding complications. However, when intravenous immunoglobulin therapy is required for a patient with ITP for thrombocytopenia, careful attention must be paid for a rapid rise in the platelet count, which may induce myocardial infarction or stroke, particularly in the elderly patients and in patients with coronary risk factors. There is no recommended dose of heparin in such cases. However, a weight-adjusted dose can be used. Caution must be exercised in low-molecular-weight heparin because of its unmonitored and long-term effects. Consequently, a double loading dose of clopidogrel (600 mg) combined with anti-platelet therapy (aspirin plus clopidogrel, 75 mg) should be prescribed. 

The choice of implanting a bare metal stent was undertaken by the operator due to desire avoid late stent thrombosis. However there is no data on the safety of drug eluting stents in circumstances like ITP [11].

This case is the first report of a young female with ITP presenting with an AMI without any cardiovascular risk factors treated with primary PCI. In conclusion, the following key issues arose from our case and review of the literature: Primary PCI is a useful, safe, and feasible therapeutic strategy in acute ST elevation myocardial infarction. Careful attention must be paid to hemostasis, therefore vessels do not injure at the time of puncture. Additionally, removing the sheath earlier and monitoring the coagulation may lead to achieve successful hemostasis. The transradial approach is particularly applicable due to the ease of compressing the puncture site. Attention must also be paid to intravenous immunoglobulin therapy in order to prevent bleeding complications. Low dose heparin (unfractionated) can be used and combined antiplatelet therapy (aspirin plus clopidogrel) should be continued.

## Figures and Tables

**Table 1:** Case reports of AMI with ITP treated with primary PCI.

Name	Age	Platelet count/*μ*L	Chronic ITP	Approach	Major Bleeding	IVIG	Steroid	Under treatment
Fuchi et al. [3]	72/F	59000	Yes	Femoral	Yes	Yes	Yes	No
Kikuchi et al. [4]	68/F	22000	No	Femoral	No	No	No	No
Kim et al. [5]	47/F	21000	Yes	Femoral	No	Yes	Yes	No
Fong et al. [6]	71/F	30000	Yes	Radial	No	Yes	No	No
Gracia et al. [7]	37/M	39000	Yes	Femoral	No	No	No	No

Current case	23/F	35000	Yes	Femoral	No	No	No	Steroids

ITP: idiopathic thrombocytopenic purpura; IVIG: intravenous immunoglobulin.
